# Cholangiopathy: Genetics, Mechanism, and Pathology

**DOI:** 10.1155/2012/950713

**Published:** 2011-09-18

**Authors:** Yasuni Nakanuma, Anthony J. Demetris, Yoshiyuki Ueno, Alberto Quaglia

**Affiliations:** ^1^Department of Human Pathology, Graduate School of Medicine, Kanazawa University, Kanazawa 920-8640, Japan; ^2^Department of Surgery, The Thomas E. Starzl Transplantation Institute, School of Medicine, University of Pittsburgh, Pittsburgh, PA 15231, USA; ^3^Department of Internal Medicine, Graduate School of Medicine, Tohoku University, Sendai 980-8575, Japan; ^4^Institute of Liver Studies, King's College Hospital, Denmark Hill, London SE5 9RS, UK

 Cholangiopathy is pathologically and pathogenetically heterogeneous and presents a broad spectrum of clinical manifestations. A majority of them are known for many years, while some are newly emerging diseases. Recent advances in biology and medicine have introduced new technologies to study the cholangiocyte biologies and physiologies and the genetics, and the pathogenesis and pathology of cholangiopathy is now being evaluated from the aspects of experimental and clinical studies. Several animal models have been developed for autoimmune and genetic cholangiopathy such as primary biliary cirrhosis and polycystic disease of the liver and biliary tract. Knowledge and understanding of these conditions have led to the development of promising therapies and novel tools to characterize these clinical conditions. 

 In this special issue of cholangiopathy, several important aspects of cholangiophany are addressed with respect to recent progress. First, in the tutorial review, the anatomy and physiology of the biliary tree and physiologic functions of biliary epithelial cells are briefly introduced for better understanding of cholangiopathy. Several important and basic mechanisms and clinicopathological features of cholangiopathy are also described. Then, Drs. M. Harada and Y. Nokanuma describe the immunological aspects of biliary tree itself and also cholangiopathy with respect to innate immunity. Several biliary diseases, particularly biliary atresia, are now being discussed from the aspect of innate immunity. Then, Dr. Zen et al. introduce a newly emerging disease of IgG4-related disease with respect to biliary tract. Many clinicians and pathologists try to categorize this disease family showing a well response to steroid therapy and involving several organs. Dr. Shimoda et al. and Dr. Sasaki et al. focused on the pathogenesis of primary biliary cirrhosis from aspects of fractalkine and cellular senescence, respectively. Dr. Shimoda et al. stress the importance, of innate and acquired immunity and Drs. K. Sasaki and Y. Nakanuma notice the participation of autophagy in the induction of cellular senescence of biliary epithelial cells. Drs. K. Tsuneyama et al. discuss the animal models of primary biliary cirrhosis which are available. Dr. Ninomiya et al. introduced a new animal model of primary biliary cirrhosis. These models will contribute to the understanding of the pathogenesis of primary biliary cirrhosis in near future. Dr. Y. Sato et al. report their accumulating data on unique animal model of Caroli's disease and discuss its molecular mechanism. This animal model resembles the autosomal-recessive polycystic disease of the kidney and liver. Lastly, Drs. G. Fava and I. Lorenzini discuss molecular pathogenesis of cholangiocarcinoma, another topic of neoplastic cholangiopathy. New biomarker and molecular targeting therapies are now rapidly evolving in this field. We are sure that this special issue will contribute to the further progress of understanding and treatment of cholangiopathy.

*Yasuni Nakanuma* *Yasuni Nakanuma* *Anthony J. Demetris* *Anthony J. Demetris* *Yoshiyuki Ueno* *Yoshiyuki Ueno* *Alberto Quaglia* *Alberto Quaglia*

